# Surgical Treatment of Hydrocephalus

**Published:** 1904-04-16

**Authors:** 


### SURGICAL TREATMENT OF HYDROCEPHALUS.

Although the hopes at one time entertained of successfully dealing with hydrocephalus by surgical methods have not been entirely fulfilled, attempts are still being made in this direction. Dr. Alfred S. Taylor[1] recently read a paper before the New York Academy of Medicine in which he records six cases of hydrocephalus where he operated with a view to making a permanent fistula between the ventricles of the brain and the sub-dural space. In support of this practice he urged that in the cases examined post-mortem there had been found almost always inflammatory changes in the choroid plexuses of the ventricles and in the ependyma covering the optic thalami, creating obstruction to the outflow of blood through the venæ Galeni and at the same time favouring increased secretion into the ventricles. A constant and important lesion was the occlusion of the natural outlet for the ventricular fluid. The operation which Dr. Taylor carried out was to turn down an osteoplastic flap about two inches in diameter, with its hinge over the base of the mastoid process. In the lower part of the dura mater thus exposed a semi-circular flap one inch in diameter was made. A slender aspirating needle was passed through the second temporo-sphenoidal lobe inward and slightly upward until it entered the ventricle. A drain of six strands of No. 2 catgut surrounded by three layers of Cargile membrane was carried into the ventricle. A sheet of Cargile membrane was interposed between the dura mater and the end of the drain, and the wound closed. Of the six cases treated in this way three died. Of the survivors one was considerably improved mentally 20 months after operation, but still was unable to support or move the enormous head. Another 11 months after operation was benefited mentally and physically, and hopes were entertained that he would become eventually a fairly normal child. The third patient lived two months after operation, and then died of some gastro-intestinal disturbance, having shown no mental improvement. Dr. Taylor lays down the following principles for the operative treatment of chronic hydrocephalus :—(1) The only feasible method is some form of internal drainage. (2) Drainage to be successful must be permanent and slow. (3) Operation must be brief, must involve the least necessary traumatism to the brain, and must be accompanied only by a small escape of ventricular fluid to avoid sudden change in the hydrostatic condition of the brain. (4) To attain the best results operation must be performed early in the disease before irreparable damage has been done to the brain structures.

[1] Med. Rec., March 19.

